# Structural basis for recognition and repair of the 3′-phosphate by NExo, a base excision DNA repair nuclease from *Neisseria meningitidis*

**DOI:** 10.1093/nar/gky934

**Published:** 2018-10-17

**Authors:** Jan Silhan, Qiyuan Zhao, Evzen Boura, Hellen Thomson, Andreas Förster, Christoph M Tang, Paul S Freemont, Geoff S Baldwin

**Affiliations:** 1 Institute of Organic Chemistry and Biochemistry of the Czech Academy of Sciences, Czech Republic; 2 Department of Life Sciences, Imperial College London, South Kensington, London SW7 2AZ, UK; 3 Dectris Ltd. Täfernweg 1, 5405 Baden-Dättwil, Switzerland; 4 Sir William Dunn School of Pathology, University of Oxford, South Parks Road, Oxford OX1 3RE, UK; 5 Department of Medicine, Imperial College London, South Kensington, London SW7 2AZ, UK

## Abstract

NExo is an enzyme from *Neisseria meningitidis* that is specialized in the removal of the 3′-phosphate and other 3′-lesions, which are potential blocks for DNA repair. NExo is a highly active DNA 3′-phosphatase, and although it is from the class II AP family it lacks AP endonuclease activity. In contrast, the NExo homologue NApe, lacks 3′-phosphatase activity but is an efficient AP endonuclease. These enzymes act together to protect the meningococcus from DNA damage arising mainly from oxidative stress and spontaneous base loss. In this work, we present crystal structures of the specialized 3′-phosphatase NExo bound to DNA in the presence and absence of a 3′-phosphate lesion. We have outlined the reaction mechanism of NExo, and using point mutations we bring mechanistic insights into the specificity of the 3′-phosphatase activity of NExo. Our data provide further insight into the molecular origins of plasticity in substrate recognition for this class of enzymes. From this we hypothesize that these specialized enzymes lead to enhanced efficiency and accuracy of DNA repair and that this is important for the biological niche occupied by this bacterium.

## INTRODUCTION

DNA is under constant stress from exogenous and endogenous damaging agents. These induce spontaneous base loss and generate vast numbers of DNA lesions that may impair DNA replication, cause mutations or DNA strand breaks, and ultimately may lead to the loss of genetic material (1,2). To counteract this potentially lethal damage, organisms from bacteria to humans have evolved a multitude of different DNA repair processes (3–6). The first line of defence is base excision repair (BER), in which the damaged base is typically removed by a DNA glycosylase to produce an abasic (AP) site (7).

Bi-functional glycosylases mainly remove lesions arising from oxidative DNA damage (e.g. thymine glycol, 5-hydroxyuracil and formamidopyrimidine). In addition to base removal, they further process AP sites and cleave the sugar-phosphodiester backbone in an elimination process (8). Two subclasses of these enzymes have been characterized and they differ in mechanism and in the reaction product. The first subclass consists of AP lyases that include human OGG1, NTH1 and bacterial Nth and generate an α,β-unsaturated aldehyde at the 3′ end via β-elimination (9–11). The other subclass of enzymes contains Nei/Fpg glycosylases with 3′-phosphate as a product formed via a β-δ elimination mechanism. This subclass includes human NEIL1 and bacterial Fpg (MutM) (12–14), among others. Together these 3′-lesions are toxic forms of DNA damage because they block the subsequent DNA polymerization step required for DNA repair (1,15). To maintain the integrity of the genome allowing further repair processes, these 3′-blocking lesions are removed by multifunctional AP endonucleases (4,16,17).

*Neisseria meningitidis* is a human pathogen that inhabits the nasopharynx and is one of the leading causes of bacterial meningitis and sepsis. Epidemic outbreaks with fatality rates of up to 50% occur mainly in sub-Saharan Africa. While massive vaccination efforts during recent decades have led to a dramatic decrease in the disease, *N. meningitidis* remains an important pathogen worldwide.

*Neisseria meningitidis* serves as a model organism thanks to its limited repertoire of DNA repair enzymes (18–21). Like many other organisms, it has two enzymes belonging to the APE1 family (22) of endonuclease (NExo and NApe). They are responsible for the elimination of 3′-lesions and the AP sites. NApe is an orthologue of human APEX1 (Ape1) and NExo, is an orthologue of *Escherichia coli* exonuclease III (ExoIII). Despite their sequence identity (27%) and high structural similarity, these nucleases have pronounced differences in activity and substrate specificity. Both NExo and NApe are exonucleases with 3′-α,β-unsaturated aldehyde phosphodiesterase activity (23). NExo was characterized as a specialized 3′-phosphatase, while NApe is an AP endonuclease, and these two activities seem to be mutually exclusive (23,24).

Our previous biochemical and genetic studies highlighted the importance of NExo in resistance to oxidative stress. A *nexo* knockout strain was very sensitive to H_2_O_2_ whilst analogous deficiency in *nape* had a milder phenotype with greater survival under this treatment (25). This observation was consistent with the exclusive processing of 3′-phosphate lesions by NExo *nexo* knockout strains did not display any observable 3′-phosphatase activity in their cellular extracts (25). Recent structural studies of APE1 family endonucleases have revealed an enzymatic mechanism for the processing of the abasic site but have not addressed the mechanism of 3′-phosphatase activity or the details behind their substrate specificity (25–29).

Here, we present structural and further biochemical characterization of NExo. To reveal the reaction mechanism of this 3′-phosphatase enzyme we determined the crystal structures of NExo in complex with a 3′-phosphate substrate and 3′-OH product DNA as well as an inactive product complex with the catalytic metal ion. In combination with a mutation analysis, this has facilitated a unique insight into the molecular basis of NExo's specificity for 3′-phosphate lesions and its mechanism of action. Mutations in the vicinity of the active site of NExo result in lowered or completely abolished 3′ phosphatase activity. Corresponding mutations of NApe mimicking NExo residues revive its latent 3′-phosphatase activity. Finally, based on our biochemical assays, we hypothesize that it is advantageous for this bacterium to maintain two enzymes because their specificity will lead to more efficient and accurate DNA repair.

## MATERIALS AND METHODS

### Mutagenesis, expression and purification of recombinant proteins

All mutants of NExo and NApe were generated by site directed mutagenesis of genes cloned into pProEx-Htb and the recombinant proteins were expressed in *E. coli* BL21(DE3) as described previously (24,25,27). In brief, protein expression was induced by addition of 0.5 mM IPTG and incubation for 6h at 22°C. The cells were pelleted by centrifugation and resuspended in 1× PBS, 500 mM NaCl and 40 mM Imidazole and 2 mM β-ME and lysed by pulse sonication. Cell lysates were clarified by centrifugation, applied on Ni Sepharose Fast Flow (GE Healthcare) and washed with identical buffer. The proteins were eluted by addition of 350 mM imidazole to the buffer. The affinity tag was removed by TEV protease digest during O/N dialysis against 20 mM Tris–HCl pH 7.5 200 mM NaCl, 10% glycerol, 2 mM β-ME. The last purification step was gel filtration on Superdex75 (GE Healthcare) in same buffers as for dialysis. Concentrated protein was flash frozen in liquid N_2_ and stored at −80°C.

### Crystallization and structure determination

DNA hairpin substrate (100 μM) with sequence 5′-ATGGCTAGCGAAGCTAG(p)-3′ (Eurogentec) (5′-ATGGTAGCGAAGCTA-3′ for Mn^2+^ product structure) were annealed by heating to 95°C for 2 min and cooled on ice using flash annealing. Complexes of NExo WT or D146N mutant with DNA were mixed with the protein at a 1.5:1 molar ratio and further purified on Superdex 200 (GE Healthcare) in 10 mM Tris–HCl pH 7.5 and 50 mM NaCl. The peak fractions containing both DNA and protein were concentrated to 250 μM. The crystals were grown at 4°C using the sitting drop where 1 μl to 1 μl of complex with reservoir containing 100 mM imidazole pH 6.6, 55 mM (NH_4_)_2_SO_4_, 10% (w/v) PEG 8000 and 16–19% (v/v) MPD, (2 mM MgCl_2_ (or MnCl_2_) for mutant protein). Mounted crystals were frozen directly in liquid nitrogen. Data were collected at the Diamond Light Source and indexed with XDS or iMosflm (30,31). Structure 2JC4 was used as a model for molecular replacement in Phaser subsequently the structures were refined using Refine and the models were manually edited in Coot, all three programs being part of Phenix package (23,32,33). Data collection and model refinement statistics are listed in Supplementary Table S1. The structure figures were generated using Pymol (http://www.pymol.org/).

### Enzymatic assays

Single-turnover experiments were carried out in reaction buffer (50 mM Tris pH 7.5, 125 mM NaCl, 1 mM EDTA, 1 mM β-ME and 0.1 mg/ml BSA) with the identical substrate and the substrates were prepared as described previously (24). Enzyme concentrations were 5 μM and substrate concentration was 100 nM. The reactions were initiated by mixing equal volumes of the enzyme with substrate at 25°C. Subsamples were taken at chosen time points typically 10 μl and quenched with 10 μl of a quenching buffer (80% formamide, 40 mM EDTA, 0.01% xylene cyanol, 0.01% bromophenol blue). The samples were separated on 20% denaturing PAGE (7 M urea, TBE). The gels were scanned on fluorescence scanner FLA-5000 (FUJI) and the resulting images were analysed and quantified using Phoretix™ 1D software. Data fitting was performed with Grafit 6 (Erithacus software), reaction models were fitted using KinTek Explorer (34).

A rapid quenched flow method was employed for nuclease assays with 3′-phosphate substrates to follow their fast reaction kinetics using a Hi-Tech RQF-63 (TGK Scientific). Reactions were performed in the same reaction buffer as above, solutions were mixed for a given time and quenched with 100 mM EDTA quenching buffer as previously described (35). Samples frozen in dry ice and all reactions were analysed as for single turnover experiments described above.

Steady-state nuclease assays were performed using AP site and its synthetic analogue (THF). Substrates were based on the previously used double stranded 50-PO_4_ (24), the native AP substrate was generated by reaction with 50 nM hUNG for 3 h, the abasic substrate was used without further treatment to avoid degradation. The THF derivative was created by direct synthesis (Eurogentec). Reactions were performed with 100 nM substrate and 0.5 nM enzyme with reaction conditions as previously described (23).

## RESULTS

### Crystal structure of NExo with its DNA substrate

**Figure 1.**
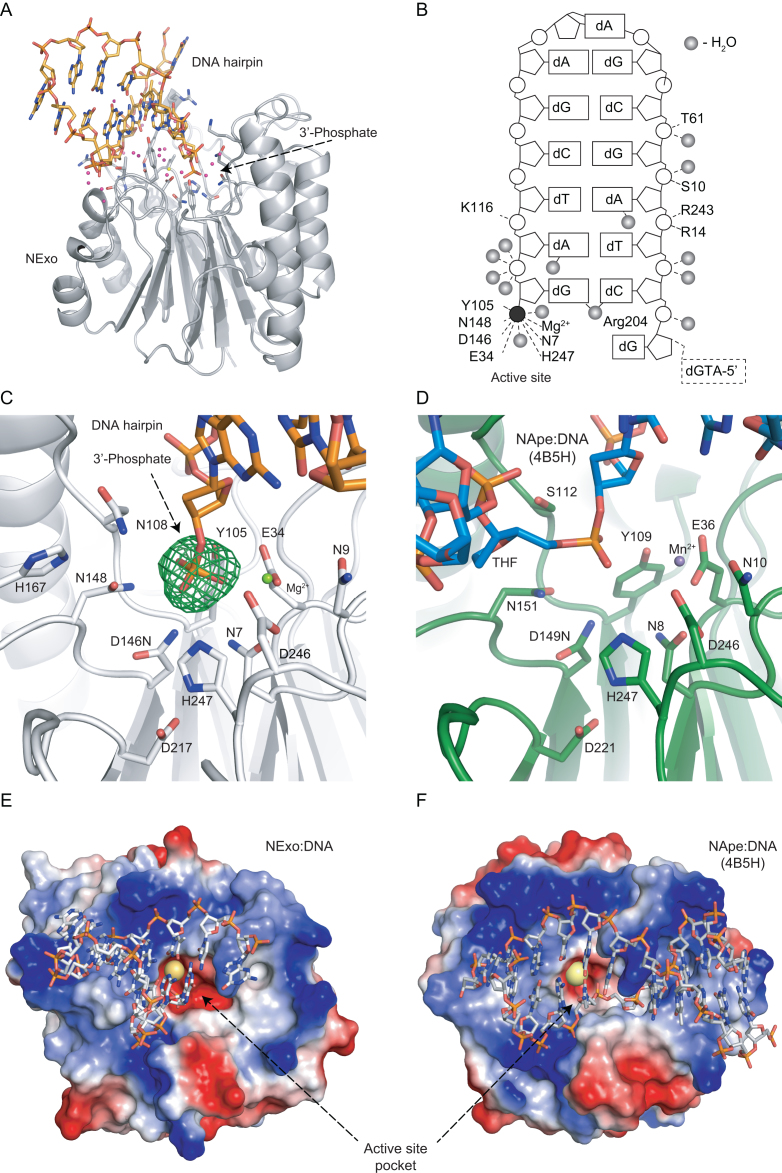
Structure of NExo-DNA (PDB ID = 6FK5). (**A**) DNA hairpin substrate (orange) with 3′-phosphate moiety bound in the NExo (grey) active site with catalytic Mg^2+^ (yellow) and water molecules of the DNA-protein interface (magenta). (**B**) Map of the interactions between DNA and NExo. Water molecules directly bridging protein and DNA are represented as grey spheres and the 3′-phosphate moiety as a black circle. R204 is intercalated between the orphan base and the base pair adjacent to the lesion. (**C**) Detail of the active site pocket of metal-bound NExo D146N (white) with 3′-phosphate DNA (orange). Coordinated Mg^2+^ (yellow) and water molecules (magenta) in the omit map (fo-fc) of electron density (green) at 5.0 sigma calculated for phosphate group excluded from calculation. (**D**) Active site pocket of NApe (green)-AP DNA (blue) complex. NApe (PDB ID code = 4B5H), (Mg^2+^ is green for NApe structure). The electrostatic surface potential of (**E**) NExo and (**F**) NApe in complex with their DNA substrates (white). Positively charged surface (blue) is located at the interface between protein and DNA. Negatively charged surface lines the active site pocket (red) accommodating a magnesium ion (yellow) and a potential exit channel for the phosphate (bottom right at the point of arrow labeling NExo's acive site).

To determine the reaction mechanism of the NExo 3′-DNA phosphatase, we solved several crystal structures of NExo with a DNA hairpin substrate containing a 3′-phosphate moiety and a product DNA lacking a 3′-phosphate group (Figure 1, Supplementary Table S1). The use of the DNA hairpin structure enabled us to capture the interaction of the enzyme with DNA; we used DNA that forms a short duplex with a 5′ overhang and a phosphate residue at the 3′-end (Figure 1A and B) (36). To facilitate direct comparison, the DNA sequence was kept as similar as possible to that in our previous NApe-DNA structures (27).

The structure of the pre-incision substrate DNA complex was solved for wild-type NExo in the absence of metal ions to prevent cleavage. In addition, a catalytic point mutant (D146N) was used to obtain the structures of both the substrate and product DNA complexes in the presence of Mg^2+^, the essential metal ion cofactor. The NExo mutation D146N is analogous to the D210N mutation which inactivates human APEX1 (37–39), and our biochemical analysis demonstrates that D146N completely abolishes activity in NExo (Supplementary Figure S1).

NExo folds into two planes of beta sheets sandwiched between two planes of alpha helices. The overall structure is similar to that of NApe (RMSD = 1.49, all Cα atoms) and other members of the AP endonuclease family such as ExoIII, APEX1 and Mth212 (23,26,38,40). NExo does not display dramatic conformational changes upon DNA binding; comparison of the substrate and product NExo complexes reveals that the oxygen (O3′) of the 3′-hydroxyl group from the terminal nucleotide is shifted by 1.3 Å (0.8 Å in the Mg^2+^ bound substrate structure) towards N108. The remainder of the protein and substrate DNA features were nearly identical (RMSD = 0.12 Å, all atoms). The position of divalent metal ion cofactors was determined for the inactive D146N in complex with substrate DNA and Mg^2+^ (Figure 1C), and for the product DNA complex with Mn^2+^ (Supplementary Figure S4). Together these structures provide a detailed insight into the interactions of NExo with 3′-phopsphate substrates, 3′-OH products and its metal ion cofactor.

Electrostatic potential maps of NExo and NApe (Figure 1E and F) reveal a negative region in the centre of the enzyme, which is the active site pocket. Here, the catalytic metal ion and the phosphate residue are accommodated. Positively charged regions surround the active site with residues essential for binding the DNA backbone. Notably, a large part of the protein interface interacts with the opposite strand of DNA. This feature highlights the basis of the preference of these enzymes for double-stranded DNA as a substrate.

A key feature of this family of enzymes is their lack of sequence specific protein-DNA interactions. Contacts between the DNA and NExo are mainly through phosphate groups on the DNA backbone, and not via nucleotides; a vast network of indirect interactions is mediated through hydrogen bonding *via* water molecules (Figure 1B). The extent of contacts mediated by water residues is greater than in the case of sequence specific DNA binding proteins, such as transcription factors (41).

### Recognition of the 3′-phosphate in the active site

A 3′-terminal phosphate moiety can originate either from a previous repair reaction by a bi-functional DNA glycosylase, or from spontaneous hydrolysis of an abasic site (9,14,16). Removal of the 3′-phosphate is essential for further repair. In the D146N structure with the 3′-phosphate substrate, we see that the scissile phosphoester bond that links the 3′-oxygen (O1P) to the phosphate is bound within the active site of NExo, and is oriented towards the mutated catalytic residue D146N. Recognition of the 3′-phosphate is mediated *via* hydrogen bonds between the 3′-phosphate and neighbouring residues N148, H247, N7 and Y105 (Figure 1B and C). Notably, the amino acids residues of the active site of the NExo 3′-phosphatase are identical to the active sites of NApe, *E. coli* Exo III and human APEX1 (Figure 1C and D) (23–25). The conservation of the active site architecture contrasts with the divergent activities that these enzymes exhibit with their different substrates; abasic lesions for NApe, 3′-α,β-unsaturated aldehyde and 3′-phosphate for NExo. Therefore, substrate discrimination cannot be governed by the active site, but must involve other aspects of molecular recognition.

### Elucidating the reaction mechanism of NExo 3′-phosphatase

**Figure 2.**
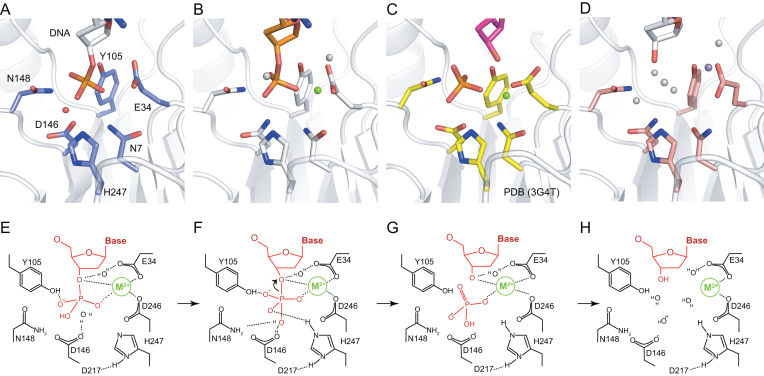
Active site during catalysis. (**A**) Detail of 3′-phosphate on DNA (white sticks) in pre-incision state, active site (blue sticks) and catalytic water molecule (red sphere) shown in NExo WT structure (PDB ID = 6FK4), (**B**) magnesium ion (green) bound in active site of point mutant substrate structure (PDB ID = 6FK5), (**C**) Phosphate group in active site pocket mimicking a leaving group (PDB ID = 3G4T) (26), (**D**) catalytic site with product DNA (white), manganese ion (violet) and water molecules (white spheres) (PDB ID = 6FKE). (**E**–**H**) Model of the catalytic mechanism of the removal of 3′-phosphate moiety (DNA in red, bivalent catalytic ion in green and active site residues of NExo in black).

Since the reaction rate of NExo is relatively fast (24), we were unable to capture snapshots of the catalytic steps. However, our structures of complexes with substrate and product have very similar organization of the active site, either with or without the metal ion cofactor. The substrate bound structure of NExo has a Mg^2+^ ion coordinated between the well-conserved D146, N7, N9 and E34, which are positioned on the leaving group side of the scissile phosphate bond (Figures 1C and 2). Therefore, the Mg^2+^ ion is well positioned to stabilize the increased negative charge of the pentavalent transition state and the displacement of negative charge onto the 3′-oxygen during the dissociative phase of the reaction (Figure 2F).

However, phosphate cleavage is not usually catalysed by a purely dissociative mechanism (42). This implies that there also needs to be activation of a water molecule positioned for in-line attack of the phosphate centre to follow an S_N_2 mechanism (Figure 2F). That the D146N mutation completely abolishes activity of the enzyme indicates a critical role for this residue in catalysis (Supplementary Figure S1). Catalytic residue D146 is positioned for an in-line attack of the phosphate centre for direct coordination and deprotonation of the attacking water (Figure 2E).

Many enzyme-catalysed phosphodiester hydrolysis reactions depend on two metal ions, where one coordinates the attacking water molecule whilst reducing its pK_a_ (43,44), and the other typically stabilizes the increased negative charge of the transition state and/or facilitates the departure of the leaving group (42). Our previous study of NApe found no evidence for a second metal ion in the active site, while the single metal ion in both NApe and NExo is directly coordinated to the scissile phosphate and is positioned on the leaving group side and is thus ideally positioned to facilitate the latter activities. In NApe it appeared that the attacking water molecule was directly coordinated for in-line S_N_2 attack by D149 with deprotonation being achieved by H247 in a proton relay with D221 (27). These key residues are directly conserved in NExo as D146, H247 and D217 (Figure 1C and D). It is therefore likely that the catalytic roles of these residues are unchanged in NExo and that it has a single metal ion mechanism analogous to NApe.

Given the very distinct differences in substrate preference between NApe and NExo despite the very high degree of structural and mechanistic conservation of their active site architecture, we decided to explore in more detail the structural determinants of substrate specificity. To this end, we examined the differences in amino acid residues that are in the vicinity of the active site to better understand the potential of these amino acid substitutions to confer AP activity with NExo, or 3′-phosphatase activity with NApe.

### NExo is a latent AP endonuclease

**Figure 3.**
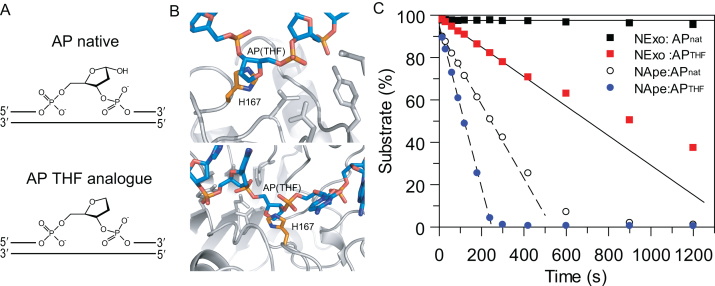
NExo discriminates AP site by 1′ hydroxyl group. (**A**) Schematic of the DNA substrate containing native abasic site (AP native) and a synthetic analogue lacking the 1′ hydroxyl group (AP THF analogue). (**B**) Model of NExo with AP site constructed by superimposing NExo (PDB ID = 6FK5) on NApe with DNA containing the AP site (PDB ID = 4B5H). NExo H167 (green) is in close contact with the AP site (DNA in blue), the active site pocket (residues outlined in white). (**C**) Steady state nuclease assay of the substrate containing either AP site or its THF analogue mixed with Nexo (squares) or NApe (circles). Plotted data are fitted to a linear equation.

In the light of detailed structures of NExo bound to its DNA substrate, we performed enzymatic assays to better understand NExo substrate specificity. Previously, we have shown that mutations of the residue H167 to glycine or serine conferred AP endonuclease activity on this enzyme (23). This residue sterically hinders the accommodation of an abasic residue in the active site (Figure 3B). Here we tested the activity of NExo with two types of AP sites, the native AP site containing a hydroxyl group at C1′ and a synthetic tetrahydrofuran analogue that lacks this hydroxyl (THF; Figure 3A). The native AP site was not cleaved by this enzyme, in line with our previous results (Figure 3C) (23). In contrast, the THF group was cleaved by the phosphodiesterase activity of NExo (Figure 3C and Supplementary Figure S3).

The product of N-glycosidic hydrolysis would result in an α-anomeric hydroxyl due to S_N_2 in-line attack of the N-glycosidic bond, whether from an enzyme-catalysed reaction or by simple hydrolysis. Comparing the NExo structure with the NApe-DNA complex, it is evident that a hydroxyl group in the α-anomer configuration of C1′ would project towards the H167 residue. The stereochemistry of this site can change through mutarotation, but this would be expected to be slow (45) compared to the enzymatic rates of abasic site cleavage. The native AP residue would thus be sterically excluded from this position, while activity assays demonstrate that some accommodation can be made for the THF analogue (Figure 3B), which lacks the 1′-hydroxyl.

To better understand the role of H167 in defining AP endonuclease activity, we tested substrates with a native AP site against NApe, *E. coli* ExoIII and the NExo mutant H167G. Reactions were performed under the single turnover conditions to define the catalytic rate of AP site cleavage (*k*_AP_), rather than the turnover rate. These data demonstrate that the native AP endonuclease enzymes ExoIII and NApe perform the cleavage step with extremely high efficiency (ExoIII *k*_AP_ = 117 ± 8 s^−1^; NApe *k*_AP_ = 413 ± 20 s^−1^; Supplementary Figure S4). As we had previously shown, NExo H167G is capable of cleaving AP sites with a catalytic rate of *k*_AP_ = 0.15 ± 0.01 (23). Therefore, the rate of the chemical hydrolysis step of AP site under single-turnover conditions was almost three thousand fold lower for NExo H167G than for that of NApe. While molecular substitutions on both the substrate and enzyme indicate that NExo has latent AP endonuclease activity, it is evident that there are further molecular determinants that define the exquisite specificity and remarkable catalytic efficiency of NApe for AP substrates.

### Determinants of 3′-phosphatase and exonuclease specificities

**Figure 4.**
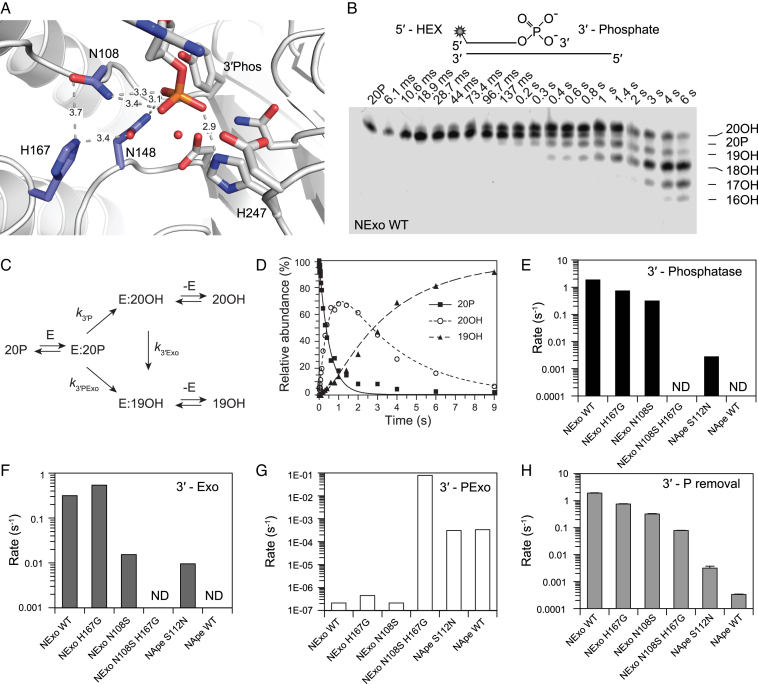
Mutations to investigate molecular mechanisms of 3′-phosphatase activity of NExo. (**A**) Detail of NExo residues different from NApe surrounding the 3′-phosphate in the active site pocket. (**B**) Schematics of the 20-PO_4_ substrate containing 3′-phosphate and 5′-fluorescent label: HEX, below single-turnover assay resolving 3′-phosphatase reaction of NExo H167G (100 nM substrate (20-PO_4_) mixed with 5 μM enzyme (quenched-flow). (**C**) Kinetic model used for data fitting. (**D**) Data from above (NExo H167G) were fitted using model (C); substrate conversion corresponds to single exponential decay. The kinetic constants from 3′-phosphatase assays were plotted in different graphs (E–G); (**E**) 3′-phosphatase, (**F**) 3′–5′ exonuclease activity and (**G**) 3′-phospho-nucleotide exonuclease activity. (**H**) Sum of 3′-phosphatase removing activities. Error bars represent SEM from three repeats; fitted rates are listed in Supplementary Figure S5B; ND donates the rate could not be determined.

To understand the origin of the distinct differences in substrate preference between NApe and NExo within the context of very high structural and mechanistic conservation, we sought to define the determinants of the 3′-phosphatase activity between these two enzymes. Systematic mutations were made to the few residues that differ within proximity of the DNA binding channel for both NExo and NApe. Target amino acids were mutated to the identity of the equivalent residue in the corresponding enzyme, resulting in NExo H167G, NExo N108S and NApe S112N (Figure 4A).

To investigate the processing of 3′-phosphate by NExo and NApe mutants we carried out single-turnover reactions with 20-PO_4_ substrate, a 20 nt oligonucleotide with 1′-HEX label and 3′-phosphate group annealed to a 50 nt long complementary strand (Figure 4 B). It was evident that the reactions displayed competing activities and were thus fitted to a kinetic model based on parallel reactions, where the 20-PO_4_ substrate can undergo 3′-phosphatase activity to yield 20-OH (*k*_3′P_), with the 20-OH product of this reaction then undergoing 3′-exonuclease activity to yield 19-OH (*k*_3′Exo_); in the parallel reaction, the 3′-phosphate in the 20-PO_4_ substrate can bypass the 3′-phosphatase reaction and undergo a direct exonuclease activity of the 3′-phospate adducted nucleotide to directly yield 19-OH (*k*_3′PExo_). It should be noted that this latter reaction is distinct from normal 3′-exonuclease activity, in which the nucleotide removed would not have a 3′-phosphate. The 19-OH (and smaller) products can thus arise from distinct parallel pathways from the 20-PO_4_ substrate (Figure 4 and Supplementary Figure S5).

Data for wild type NExo fitted well to the kinetic model described above, exhibiting a fast 3′-phosphatase reaction (*k*_3′P_, 1.9 s^−1^) coupled with a reasonably strong 3′-exonuclease activity (*k*_3′Exo_, 0.31 s^−1^) while direct exonuclease activity of the 20-PO_4_ substrate was insignificant (*k*_3′PExo_, 2.1E–07 s^−1^). This analysis clearly demonstrates that NExo processes the substrate almost exclusively via a two-step mechanism, with the first step being the removal of the 3′-phosphate, leading to significant accumulation of the 20-OH intermediate (Figure 4B and D). Examination of the NExo N108S and H167G mutants revealed that both had a reduction in the rate of 3′-phosphatase activity (*k*_3′P_; Figure 4E, Supplementary Figure S6), while H167G had a minor increase and N108S a minor decrease in 3′-exonuclease activity (*k*_3′Exo_; Figure 4F, Supplementary Figure S6): in both instances the direct 3′-phosphate exonuclease activity remained insignificant (*k*_3′PExo_; Figure 4G, Supplementary Figure S6).

Examination of NApe was consistent with our previous data with the absence of 3′-phosphatase activity. Degradation of the 20-PO_4_ substrate arose solely from direct exonuclease activity of the 3′-phospate adducted nucleotide, at a very low rate (*k*_3′PExo_, 3.3E–04 s^−1^), although still four orders of magnitude higher than that of NExo. Intriguingly the NApe S112N mutation, while exhibiting a similar level of 3′-phosphate exonuclease activity clearly showed 3′-phosphatase activity (*k*_3′P_, 2.8E–03 s^−1^; Figure 4, Supplementary Figures S5 and S6). The excision of a 3′-phospate monoester is not directly analogous to the phosphodiester cleavage in 3′-exonuclease activity since the 3′-phosphate contains additional negative charge that has to be further compensated in the pentavalent transition state, making it a significantly more challenging reaction to catalyse. The enhanced 3′-phosphatase activity in S112N NApe and the depletion of this activity in N108S NExo suggest that the amine of this residue may assist in transition state stabilization. The coordination of two amine groups from N108 and N148 to the scissile phosphate may thus have a role in offsetting the enhanced negative charge in the transition state of 3′-phosphate monoester cleavage. It is also similar to the coordination observed in the reaction of alkaline phosphatase with phosphate monoesters, where R166 directly coordinates the scissile phosphate via two amine groups. Furthermore, H167 in NExo may lead to a local proton relay or p*K*_a_ shift in concert with N148 and N108 to provide the additional catalytic power required to remove the 3′-phosphate (Figure 4A).

Based on this and the previous abasic DNA cleavage (Figure 3C) (23), it is clear that the amino acids H167 and N108 in NExo and their equivalents in NApe have a role in discriminating between 3′-phosphate and abasic DNA lesions. To further evaluate this, we examined the NExo N108S/H167G double mutant. In single turnover experiments with 20-PO_4_ DNA a dramatic difference was observed. Similarly as for wild type NApe we were unable to detect any 3′-phosphatase activity. The removal of the 3′-phosphate moiety was carried out by a significant increase in direct 3′-phosphate exonuclease activity (*k*_3′PExo_, 0.08 s^−1^) that was >100-fold higher than the wild type enzyme, and S112N NApe. This change in activity profile also necessitated fitting the data to an alternative model since the fast exonuclease rate resulted in dissociation of the substrate into single-stranded DNA (Figure 4, Supplementary Figure S6).

Our data suggest that no single point mutation of NExo or NApe can reinstate wild type levels of activity to non-cognate substrates. However, surprisingly simple mutations did confer latent activities, including 3′-phosphatase activity to NApe via the S112N mutation and AP endonuclease activity to NExo via H167G. Intriguingly, the NExo N108S H167G double mutant introduced significant levels of direct 3′-phosphate exonuclease activity, which is not cognate to any of the AP endonuclease family of enzymes that we have tested. A 3′-phosphate adducted nucleotide is an unusual lesion since although it is a conventional phosphodiester linkage that is hydrolysed during exonuclease activity, the terminal 3′-phosphate carries an additional negative charge and has to be accommodated in the DNA binding channel. In the same manner that H167 excludes an abasic site, it is also likely to exclude the 3′-phosphate, so the H167G provides a steric advantage, while the N108S mutation is better adapted to exonuclease activity; thus, the combination of N108S and H167G mutations provides an unusual proclivity toward 3′-phosphate exonuclease activity not observed in the wild type enzymes.

## DISCUSSION

NExo is one of two enzymes from *Neisseria meningitidis* belonging to the APE1 family of AP endonucleases. Unusually it lacks AP endonuclease activity, but has 3′-phosphatase activity that is lacking in the second enzyme NApe (24). This work aimed to gain mechanistic insights into the basis of the differing substrate specificities of these two enzymes. We have captured crystal structures of NExo with its 3′-phosphate substrate and 3′-hydroxyl product in double stranded DNA complexes, both with and without the metal ion cofactor. These structures reveal a remarkable degree of both structural and mechanistic conservation between NExo and NApe, with their active sites being nearly identical, despite significant differences in substrate specificity.

Our kinetic analysis of mutant enzyme demonstrated that simple point mutations could switch on and off selected enzymatic activities. While these mutants do not possess the kinetic characteristics of fully evolved enzymes, this plasticity of function is indicative of evolutionary specialization within the APE1 family of enzymes. Further, plasticity of function in this enzyme family is evident from the enhancement of a non-cognate 3′-phosophate exonuclease activity in NExo N108S/H167G (Figure 4).

An interesting comparison can be made with *E. coli* alkaline phosphatase, where significant mechanistic studies of have been conducted to compare the reactivity of phosphate monoester and diester substrates that are analogous, respectively, to the 3-phosphate and phosphodiester cleavage reactions studied here (46). Alkaline phosphatase monester hydrolysis proceeds through a looser, more dissociative transition state than diester hydrolysis. The *E. coli* alkaline phosphatase is sufficiently plastic to accommodate both transition states. Our studies with NExo and NApe demonstrate that this is not the case for this enzyme pair. While this can be partly reconciled by steric constraints, it is evident from the enhanced diesterase activity of NExo N108S/H167G that mutational divergence can clearly lead to different mechanistic pathways, presumably through active site optimization for different transition states.

*Escherichia coli* ExoIII is an orthologue of NExo, it exhibits significant plasticity in substrate recognition and has activity across all substrate specificities observed for this class of enzyme (AP endonuclease, 3′-phosphatase, phosphodiesterase, 3′–5′ exonuclease). ExoIII is basally expressed in *E. coli* but is also controlled by RpoS (47,48), a general stress response regulator (49). The ability of ExoIII to act during a general response to DNA damage may be due to its enzymatic dexterity in effectively processing numerous lesions, although this may be at some fitness cost; for instance it is known to antagonize double-strand break repair by RecBCD (50).

*Neisseria meningitidis* appears to adopt a different strategy: although lacking an SOS response (51), it has two constitutively expressed APE1 family enzymes (23) with distinct specialization (24,25,27). Our current and previous results indicate that in the meningococcus they may have evolved side by side. As an obligate commensal *N. meningitidis* has to survive routine exposure to the oxidative burst of macrophages during cycles of infection. This has been achieved by the presence and separate evolution of two homologous nucleases with well-defined and highly specialized DNA repair functions, probably with fewer side reactions than a single broad-activity enzyme like ExoIII, and hence leading to more efficient and more accurate DNA repair. Therefore, the maintenance of two highly specialized enzymes may be an essential requirement for the biological niche that *N. meningitidis* occupies.

## DATA AVAILABILITY

Structures were deposited in the Protein Data Bank under accession numbers: 6FK4, 6FK5 and 6FKE.

## Supplementary Material

Supplementary Data
