# Supplementary material for: Structural basis for recognition and repair of the 3′-phosphate by NExo, a base excision DNA repair nuclease from Neisseria meningitidis
Source: Nucleic Acids Res. 2018 Oct 17;46(22):11980–9. doi: 10.1093/nar/gky934 (PMC6294502; doi:10.1093/nar/gky934)
Supplement: Supplementary Data [file gky934_supplemental_files.pdf]

Supplementary material

Structural basis for recognition and repair of the 3′ phosphate by NExo, base excision DNA repair nuclease from *Neisseria meningitidis*

Jan Silhan[1,*], Qiyuan Zhao[2], Evzen Boura[1], Hellen Thomson[2], Andreas Förster[3], Christoph M Tang[4,*], Paul S. Freemont[5,*], Geoff S. Baldwin[6,*]

1. Institute of Organic Chemistry and Biochemistry of the Czech Academy of Sciences
2. Previously of: Department of Life Sciences, Imperial College London, South Kensington, London SW7 2AZ
3. Dectris Ltd. Täfernweg 1, 5405 Baden-Dättwil, Switzerland
4. Sir William Dunn School of Pathology, University of Oxford, South Parks Road, Oxford, OX1 3RE
5. Department of Medicine, Imperial College London, South Kensington, London SW7 2AZ
6. Department of Life Sciences, Imperial College London, South Kensington, London SW7 2AZ

* To whom correspondence should be addressed: g.baldwin@imperial.ac.uk; p.freemont@imperial.ac.uk; christoph.tang@path.ox.ac.uk; silhan@uochb.cas.cz;

Supplementary Table 1: Data collection and refinement statistics

Crystal structure	NExo WT: DNA Sub	NExo D146N: DNA Sub Mg^{2+}	NExo D146N: DNA Product Mn^{2+}
PDB ID	6FK4	6FK5	6FKE
Diffraction source	Diamond I-04-1	Diamond I-24	Diamond I-24
Wavelength (Å)	0.91730	0.96860	0.96860
Space group	P 43 21 2	P 43 21 2	P 43 21 2
Cell dimensions			
a, b, c (Å)	59.2 59.2 292.1	58.9 58.9 292.3	58.9 58.9 292.5
α, β, γ (°)	90, 90, 90	90, 90, 90	90, 90, 90
Resolution range (Å)	41.41 - 2.32 (2.40 - 2.32)	50.43 - 2.02 (2.08 - 2.02)	38.32 - 2.15 (2.23 - 2.15)
Total no. of reflections	157389 (15668)	70434 (6651)	185196 (18217)
No. of unique reflections	24224 (2282)	35871 (3478)	29178 (2803)
Multiplicity	6.5 (6.8)	2.0 (1.9)	6.3(6.5)
Completeness (%)	99 (97)	99 (100)	99.1 (98.5)
Mean I/σ(I)	14.18 (1.78)	7.40 (1.77)	14.10 (2.23)
R-meas (%)	10.47 (62.52)	5.81 (44.42)	7.79 (56.51)
CC1/2	0.993 (0.961)	0.996 (0.91)	0.999 (0.957)
CC*	0.998 (0.99)	0.999 (0.976)	1 (0.989)
R-work (%)	19.85 (30.15)	23.02 (37.82)	20.00 (32.76)
R-free (%)	23.00 (37.71)	25.53 (36.96)	22.42 (36.29)
Number of non-H atoms	2451	2499	2491
RMSD			
Bonds (Å)	0.003	0.006	0.010
Angles (°)	0.62	0.89	1.21
Ramachandran favored/ outliers (%)	96/0	95/0.4	96.5/0
Average B-factor ($Å^2$)	66.43	49.63	50.46

Statistics for the highest-resolution shell are shown in parentheses.

Supplementary Figure 1

A

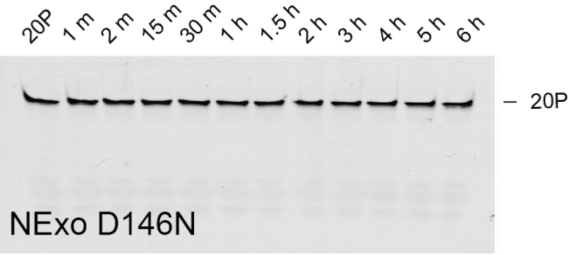

Single-turnover assays of 3′ phosphatase activity. Enzymatic reaction of the inactive mutant NExo D146 resolved on 20% denaturating PAGE gel. HEX labelled 20P DNA substrate was mixed with an enzyme and subsamples were quenched at indicated time points.

Supplementary Figure 2

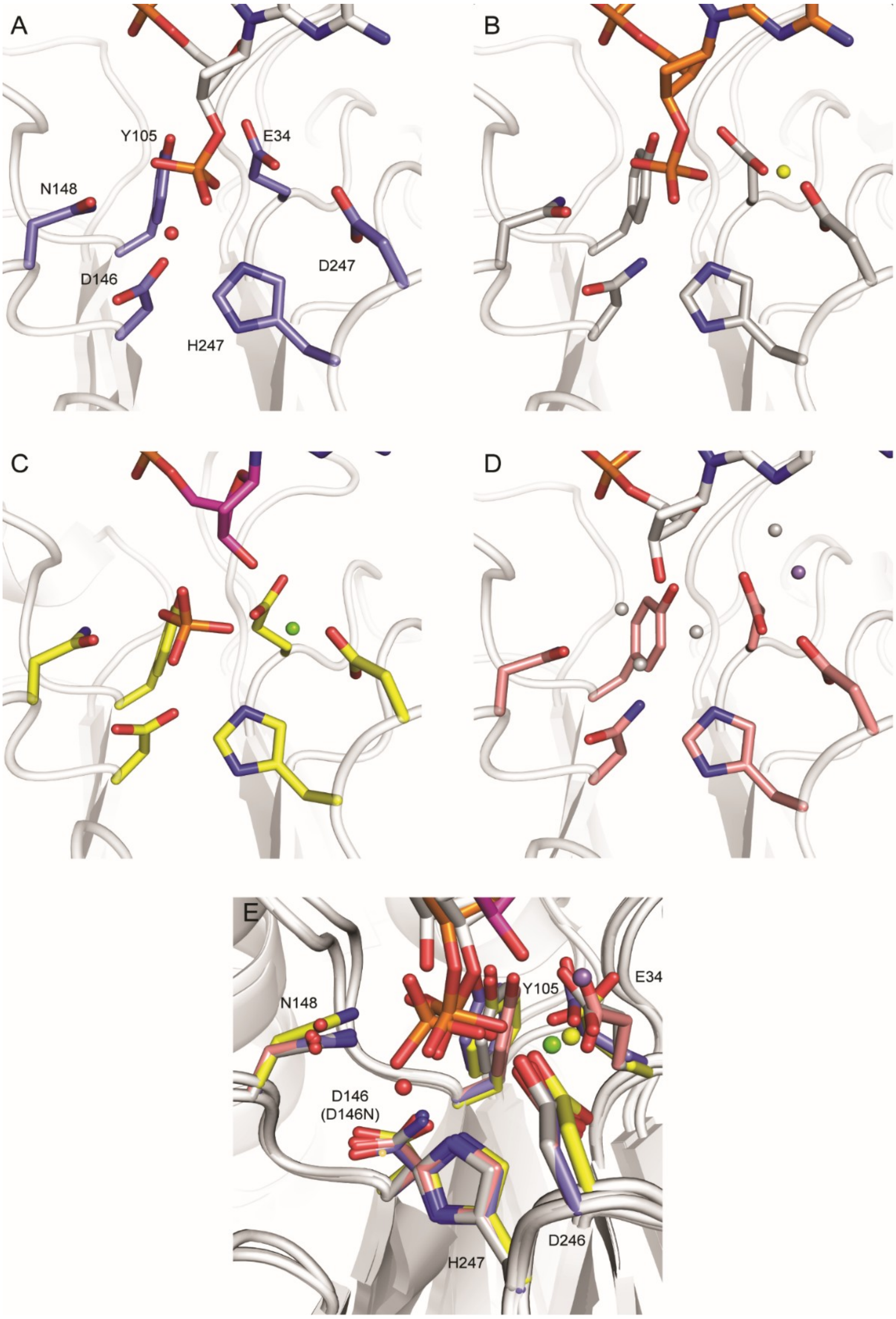

Overlay of structures outlining different reaction stages. (A) Detail of 3′ phosphate on DNA (white sticks) in pre-incision state, active site (blue sticks) and catalytic water molecule (red sphere) shown in NExo WT structure (PDB ID = 6FK4), (B) magnesium ion (yellow) bound in active site of point mutant substrate structure (PDB ID = 6FK5), (C) published structure of ortholog Mth212 (white and yellow) with DNA (purple), (PDB ID = 3G4T[1]) coordinating phosphate from the solvent and Mg^{2+}(green) in the active site pocket (D) active site pocket containing Mn^{2+} (purple) water molecules (white spheres) reside in position and arrangement resembling oxygen atoms from phosphate group in product structure (PDB ID = 6FKE). (F) An overlay of alignment of structures in panels A-D, (residues and DNA chains are represented in identical colours as for individual structures in panels above)

Supplementary Figure 3

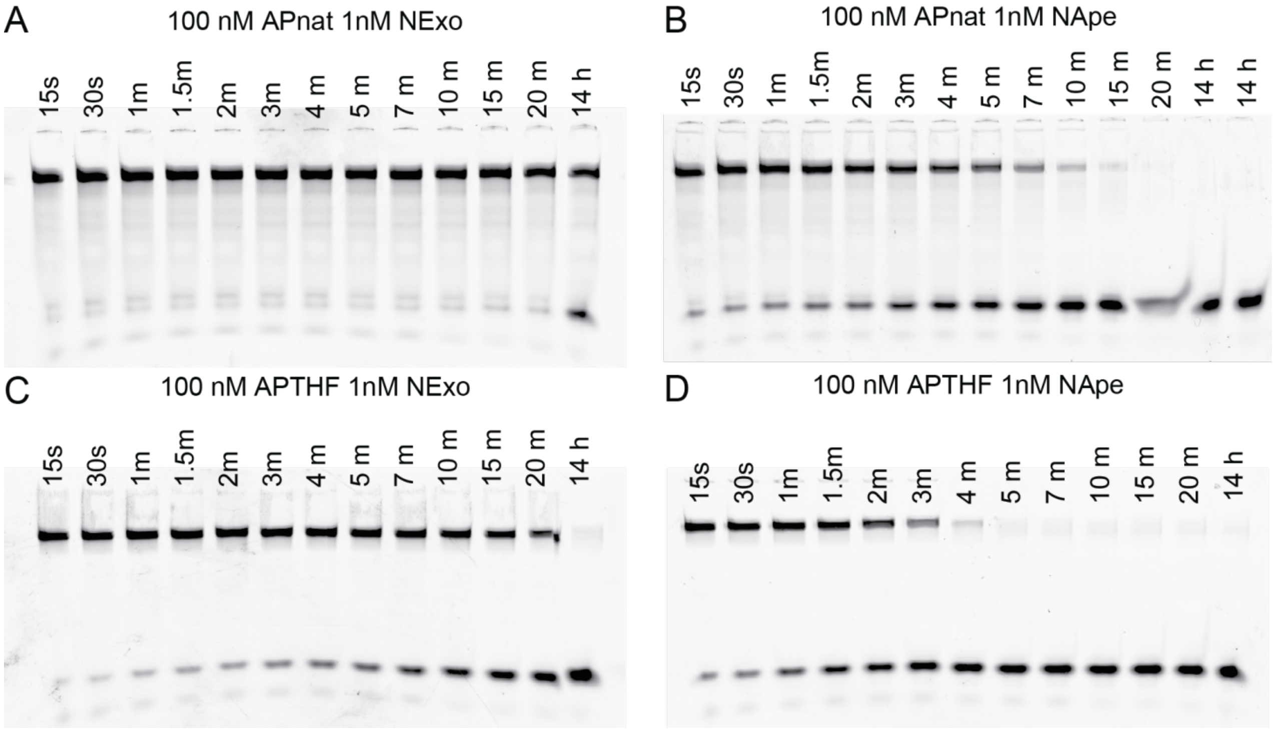

AP endonuclease assay comparing native AP site and it synthetic (THF) analogue. Denaturating 20% PAGE gel; 100 nM native AP site DNA substrate was incubated with 1 nM (A) NExo and (B) NApe and quenched in indicated time points. Identical reaction was set up with 100 nM DNA substrate containing THF AP analogue and 1 nM (C) NExo and (D) NApe.

Supplementary Figure 4

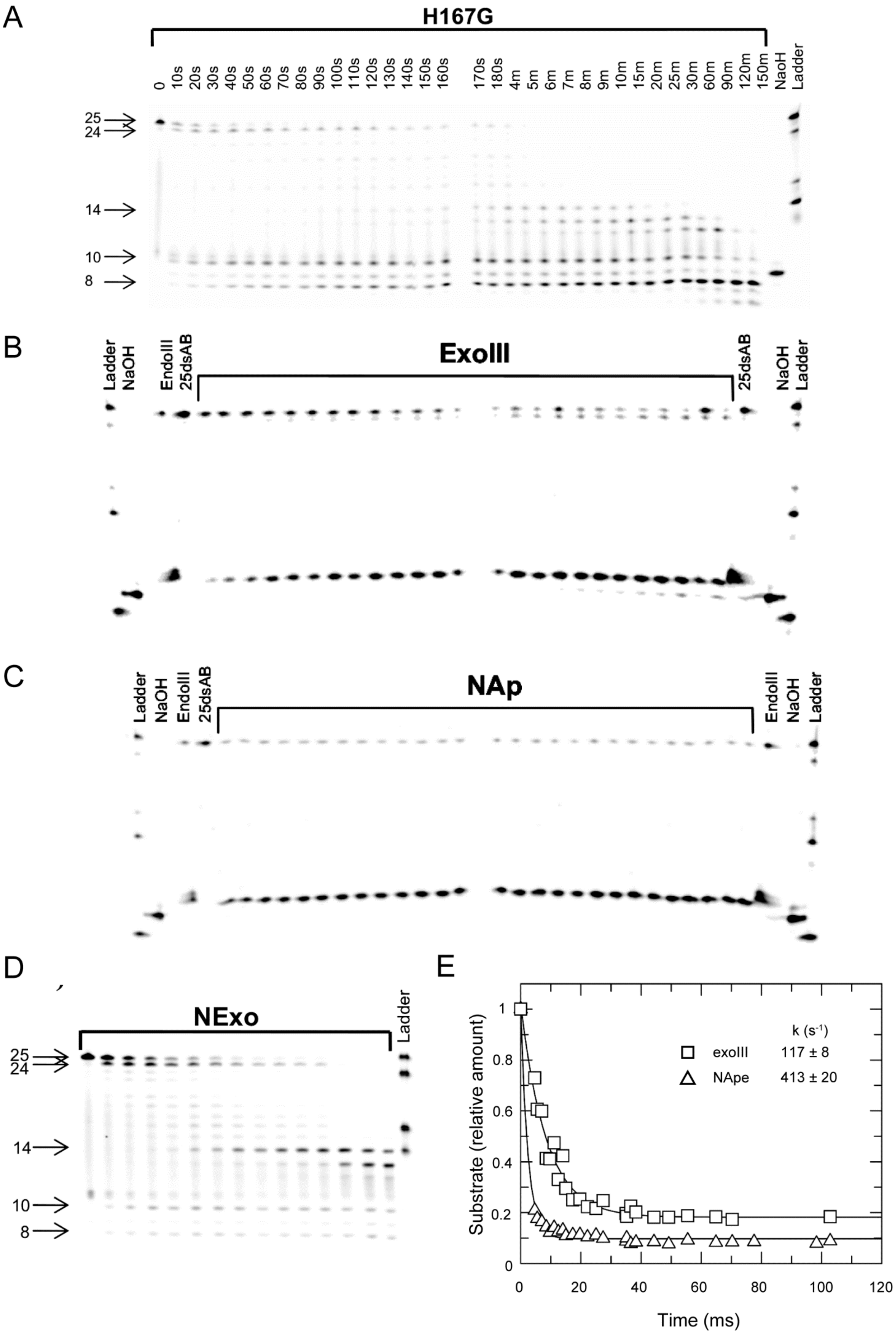

AP endonuclease assays under single-turnover conditions. Comparison of NExo His167Gly, NApe, ExoIII and NExo. 25dsAB DNA containing uracil was treated with Udg to generate AP sites, 100 nM of this substrate was reacted with 500 nM of enzyme and aliquots in different time points were taken and analysed on the gel, (A) NExo His167G, (B) ExoIII, (C) NApe and (D) NExo WT. Resulting data were plotted into the graph and fitted with single-exponential decay. Its rate constant represents catalytic rate of the reaction. Enzymes were purified as described previously[2].

Supplementary Figure 5

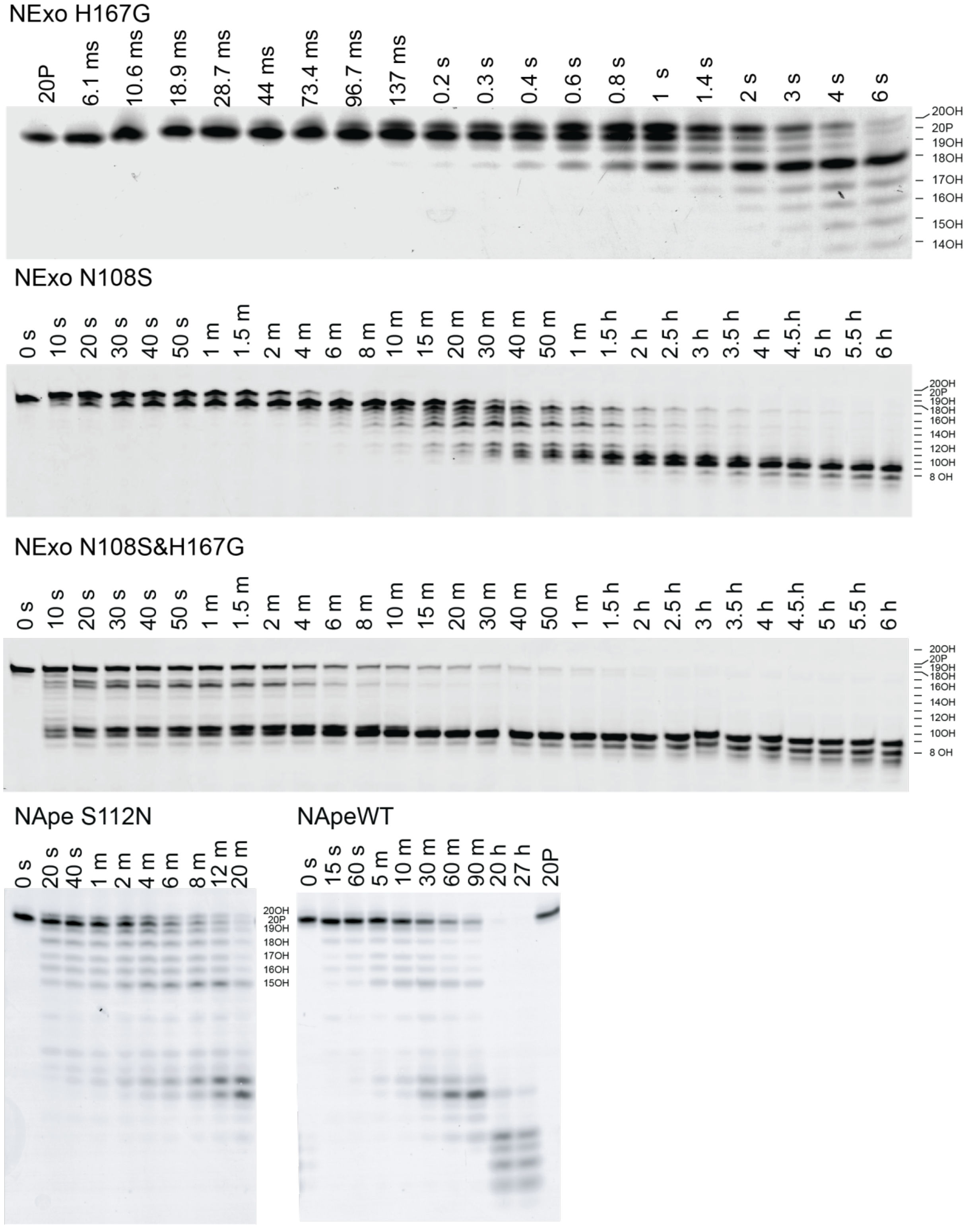

Single-turnover assays of 3′ phosphatase activity. Gels of single turnover reaction of NExo and NApe mutants as marked above the gels. NExo H167G, N108S and NExo double-mutant N108S&H167G. No 20OH band corresponding to the product formation (band above 20P substrate band) is clearly visible as in case of double mutant. (For gel figures and for direct comparison of WT and inactive form of NExo please refer to Fig. 4 and Fig. S1.)

Supplementary Figure 6

A

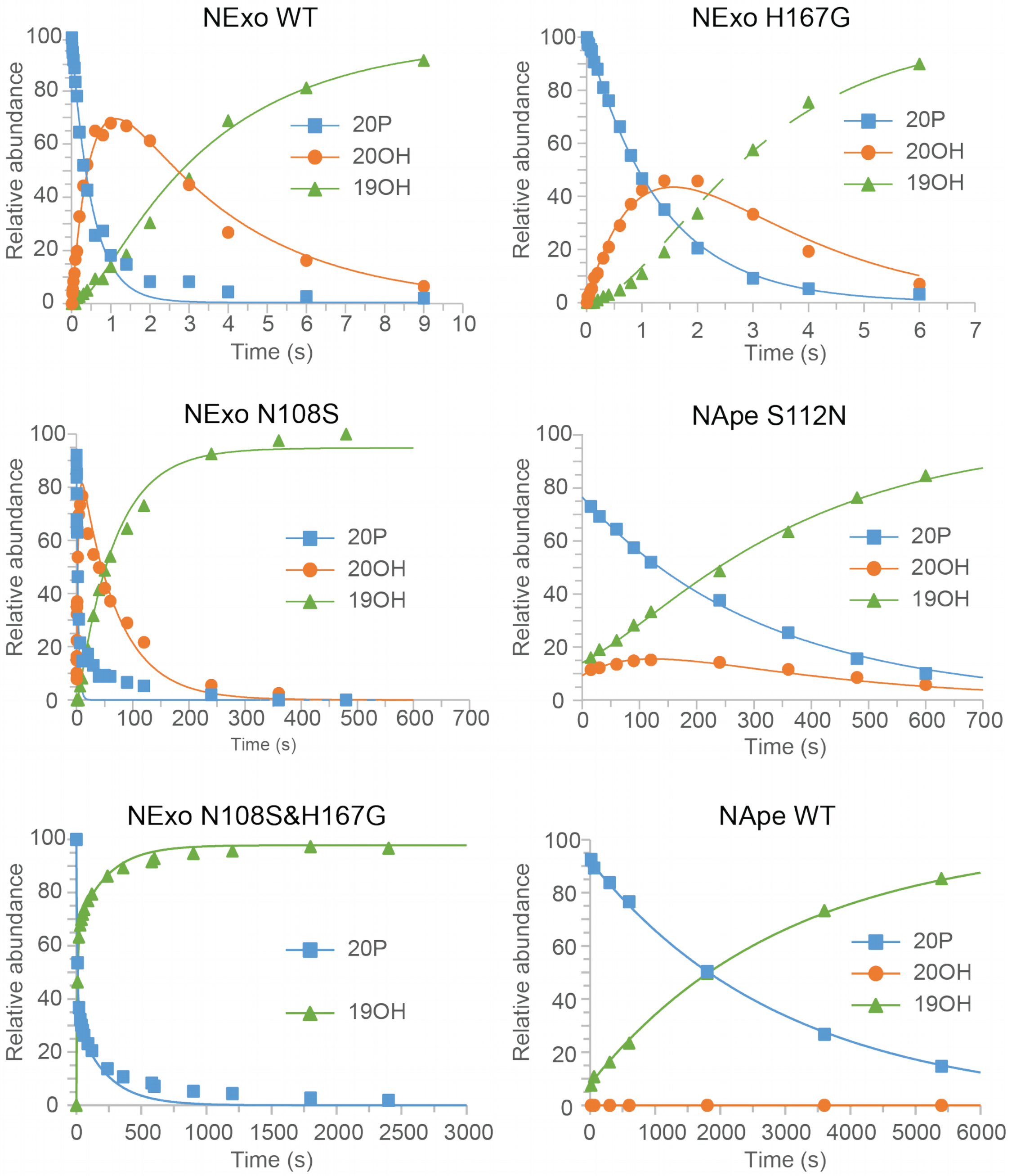

B

Reaction rates

k (s^{-1})

	$k_{3'P}$	$k_{3'PExo}$	$k_{3'OHExo}$	$k_{3'ssPExo}$
NExo WT	1.92	2.11E-07	0.314	
NExo H167G	0.754	4.46E-07	0.539	
NExo N108S	0.322	2.11E-07	0.0153	
NExo N108S H167G	ND	0.0788	ND	0.0043
NApe WT	0	0.000334	ND	
NApe S112N	0.00284	0.000309	0.00953	

Single-turnover rates of 3′ phosphatase assays. (A) Quantification and fit of reactions for individual proteins and their mutants. Note the difference in scale. (B) Individual rates are listed in a table. ND stands for insufficient data where the rates were not determined The parallel kinetic

scheme described for analysis of the 20P substrate could not be fitted to the data from NExo N108S H167G because there was no data for the 20OH intermediate suggesting that 3′-phosphatase activity was not present; furthermore the accumulation of 19OH and smaller products was biphasic and could not be fitted to the single rate of the direct exonuclease pathway ($k_{3'PExo}$). The high level of direct exonuclease activity indicated that all substrate was being processed via this route, but also the second unlabelled strand was also being processed by 3′-exonuclease activity. The shortness of the DNA substrate (20 bp) meant that only one strand could be processed at a time. As a result both labelled and unlabelled strands were being processed during the first few time points leading to disassociation of DNA duplex. This would therefore lead to approximately half of the labelled DNA being processed as a single-stranded substrate. A revised kinetic scheme based on processing of both ds and ss DNA was therefore devised (Supp Fig. 7), which included a 3′ exonuclease rate for the labelled single-stranded DNA ($k_{3'SSPEx}$ 0.0043 s^{-1}), which led to a complete fit of the data.

Supplementary Figure 7

A

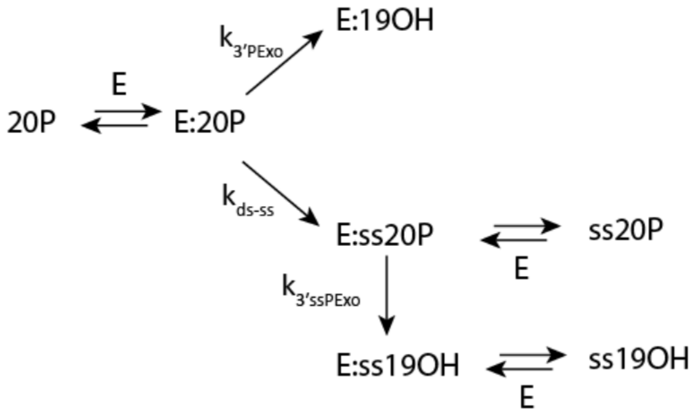

Kinetic scheme used for the fitting of the NExo N108S H167G 3′P exonuclease activity. The ds DNA substrate can be processed by direct exonuclease excision of the 3′P adducted DNA ($k_{3'PExo}$), but the enhanced exonuclease activity of this mutant also means that the ds DNA substrate can be processed to ss DNA substrate via exonuclease activity of the unlabelled DNA strand ($k_{ds\text{-}ss}$) to yield the E:ss20P complex, which can then be processed by single strand exonuclease activity ($k_{3'ssPExo}$).

References

1. Lakomek, K., Dickmanns, A., Ciirdaeva, E., Schomacher, L. & Ficner, R. Crystal Structure Analysis of DNA Uridine Endonuclease Mth212 Bound to DNA. *J Mol Biol* **399**, 604-617 (2010).
2. Carpenter, E.P. et al. AP endonuclease paralogues with distinct activities in DNA repair and bacterial pathogenesis. *Embo J* **26**, 1363-1372 (2007).
